# Application of read-across methods as a framework for the estimation of emissions from chemical processes

**DOI:** 10.3934/ctr.2023018

**Published:** 2023-12-28

**Authors:** Sudhakar Takkellapati, Michael A. Gonzalez

**Affiliations:** US Environmental Protection Agency, Office of Research and Development, Center for Environmental Solutions and Emergency Response, Land Remediation and Technology Division, Environmental Decision Analytics Branch, 26 W. Martin Luther King Dr., Cincinnati, OH 45268, USA

**Keywords:** read-across, chemical process emissions, source chemical, target chemical, analogue chemical, chemical family, category of chemicals, structural similarity

## Abstract

The read-across method is a popular data gap filling technique with developed application for multiple purposes, including regulatory. Within the US Environmental Protection Agency’s (US EPA) New Chemicals Program under Toxic Substances Control Act (TSCA), read-across has been widely used, as well as within technical guidance published by the Organization for Economic Co-operation and Development, the European Chemicals Agency, and the European Center for Ecotoxicology and Toxicology of Chemicals for filling chemical toxicity data gaps. Under the TSCA New Chemicals Review Program, US EPA is tasked with reviewing proposed new chemical applications prior to commencing commercial manufacturing within or importing into the United States. The primary goal of this review is to identify any unreasonable human health and environmental risks, arising from environmental releases/emissions during manufacturing and the resulting exposure from these environmental releases. The authors propose the application of read-across techniques for the development and use of a framework for estimating the emissions arising during the chemical manufacturing process. This methodology is to utilize available emissions data from a structurally similar analogue chemical or a group of structurally similar chemicals in a chemical family taking into consideration their physicochemical properties under specified chemical process unit operations and conditions. This framework is also designed to apply existing knowledge of read-across principles previously utilized in toxicity estimation for an analogue or category of chemicals and introduced and extended with a concurrent case study.

## 1. Introduction

In February 2022, the US EPA launched an improved effort under the Toxic Substances Control Act (TSCA) to modernize the review process and introduce innovative science into the review of new chemicals before they can enter the marketplace. Through this effort, the Office of Chemical Safety and Pollution Prevention (OCSPP) is proposing to develop and implement a multi-year collaborative research program in partnership with the Agency’s Office of Research and Development and other federal entities focused on approaches for performing risk assessments on new chemical substances under TSCA. One component of this collaboration is to investigate extension of OCSPP’s current approach by applying read-across methods, which use data from structurally similar chemicals with known risks to determine potential risks from new chemicals that are structurally analogous. This approach is anticipated to increase the efficiency and timeliness of new chemical reviews and promoting the use of the best available data with the end goal of protecting human health and the environment. Simply put, this will facilitate the new chemicals program tracking of decisions over time and evaluating consistency within and across chemistries.

In the manufacturing phase of a chemical, as well as in the use and application phases, there are attributed environmental releases and emissions. The estimation and quantification of these emissions at different life cycle stages are one of the key factors in exposure assessment. It is critical to have quantitative knowledge of chemical releases into the environment to fully understand and predict (quantify) human exposure. Although significant efforts have been directed to characterize and screen organic chemicals for hazardous properties, relatively less emphasis is placed on estimating emissions throughout the life cycle [1]. Chemical screening for potential hazard to the environment, as well as to human health, has relied on characteristics such as persistence, bioaccumulation, toxicity, and transport potential which are relative to the chemical itself. However, the assessment of chemical exposures and potential risk additionally requires information on emissions attributed to the manufacture, use, and end-of-life stage associated with the chemical [1]. Quantification of emissions at various life cycle stages of chemicals and chemical containing products is the starting point of fully encapsulating exposure assessment and is the key to any modeling effort aimed at deriving predicted environmental concentrations in air, water, and soil. Unfortunately, due to the complex nature of the chemical emission patterns, it is often difficult to fully and accurately quantify the emissions. These uncertainties in estimation of an emissions inventory will lead to uncertainties in the ensuing exposure assessment [2]. Thus, it is critical to have an element of measured environmental emission data to have a reliable predicted environmental concentration of a chemical to provide a dependable exposure assessment, and eventual risk determination. It is imperative to improve the estimation of life cycle emissions to advance exposure and risk assessment of chemicals in or proposed for commerce and provide more information to allow for improved decision support.

A review of the literature identified four general techniques that exist to estimate chemical emissions. (1) Direct measurement; (2) Use of an emission factor; (3) Mass balance and (4) engineering calculations [3]. Direct measurement is a technique used to measure directly at the emission source itself either by using stack tests, an emissions monitoring system, or a similar monitoring technique. The data obtained by direct measurement methods is more accurate since it is obtained through experimental measurement at the source of emission instead of an estimation. An emission factor is a representative value that attempts to relate the quantity of a pollutant emitted from a chemical from an industrial activity, and quite often is used for estimating emissions [4]. However, since emission factors are used in a correlation sense, they generally overestimate emissions. The mass balance method is based on calculations where one assumes that the inputs and outputs of a process are equal and directly linear. Thus, this method can also lead to over estimation of emissions. Finally, engineering calculations are developed based on process knowledge and expert judgement to estimate emissions [5]. Although direct measurements provide the most accurate and representable environmental release information, this method is the most expensive and time-consuming to implement and is highly unlikely to be completed for every chemical process unit operation. Thus, if environmental emission and release data for a set of analogous chemicals in a category are available, one can apply a read-across methodology to begin to fill the data gaps either by interpolation or extrapolation.

To increase the need for being able to provide quantification of emissions to the manufacturing, use, and end-of-life stages is the recently enacted Sustainable Chemistry Research and Development Act as part of the must-pass National Defense Authorization Act for Fiscal Year 2021. The act has two main elements. One is the formation of an interagency committee to determine the baseline status of sustainable chemistry activities across the federal government and the US economy [6]. This baseline will be used to measure the progress and effectiveness of activities envisioned under the act. The other element is organizing coordinated support for federal efforts in sustainable chemistry, including research and development, technology transfer, commercialization, and education and training. In short, the bill creates a federal government-wide effort that could enable the United States to lead globally in the innovation, commercialization, and adoption of safer, more sustainable chemicals and materials in the future. The coupling of these two efforts places an increased emphasis on a proactive approach to the evaluation of chemicals prior to entering commerce.

This innovative research contribution demonstrates the extended application of read-across processes by providing a methodology and ensuing framework to utilize this technique for estimating chemical process emission data gaps by utilizing the physicochemical properties coupled with available emission data obtained by direct measurements for a set of chemicals in a specified manufacturing process. The application of read-across can also be utilized to fill in the data gaps of certain chemical properties, and these chemical properties may also be obtained from EPI suite [7] or other chemical property estimators such as Toxicity Estimation Software Tool [8] etc. The aim of this methodology would be to construct a framework (database) with all the available sources of data for chemicals and their emissions in various processes, and to then fill the data gaps using machine learning processes whereby machine learning serves as a tool for data extraction, data pattern recognition, and data prediction.

## 2. Methodology

The read-across process uses available data, for example, of one chemical structure being utilized to correlate the relevance of that information to a structurally analogous chemical. Thus, read-across is regarded for filling data gaps to aid in predicting endpoint information for a target substance by using the known data for the same endpoint of a source substance [9,10]. Read-across is primarily developed as an alternate methodology for animal testing to provide supporting data for toxicity and hazards of a specific chemical. Currently, a variety of scenarios are used to establish similarity, such as structural or mechanistic analogues, the formation of common metabolites, and a physicochemical property, etc. [11–15]. Chemical structure similarity, reactivity, and physicochemical property similarities are the main characteristics employed to justify the use of a read-across approach. The read-across toolbox is progressively expanding in the development of databases, tools to establish structural similarity and algorithms for quantitative read-across methods. The use of appropriate cheminformatics is very critical in managing the data and allowing the predictivity of a read-across approach while minimizing its uncertainty [16].

The terms analogue approach and category approach are used to describe techniques for grouping of chemicals that have similar structure, attributes, properties, or characteristics. The analogue approach refers to the grouping of a target and source analogue chemicals together, whereas the category approach refers to the grouping of a target with at least two or more source chemicals [10]. The target chemical is referred to as the chemical of interest, and the source chemical refers to structurally similar chemicals with respect to the target chemical [9].

In the read-across analogue approach data from a source chemical is used to make an assessment concerning the relevance of that information onto a target chemical. This process results in one or more properties of a given chemical (source) being inferred through comparison of that chemical with another chemical of similar molecular structure (target) and physicochemical properties, for which the properties of interest are known. This approach can be used to evaluate physicochemical properties, toxicity, environmental fate, and eco-toxicity [10].

The read-across approach using either method can be performed qualitatively or quantitatively. The qualitative read-across process involves the identification of a chemical sub-structure that is common to the two analogous substances and the assumption that the presence/or absence of a property/activity for a substance can be inferred from the presence/or absence of the same property/activity for an analogous substance. This assumption implies that analogues behave qualitatively similarly and is usually the result of an expert judgement evaluation. Quantitative read-across involves the identification of a chemical sub-structure that is common to the two analogous substances, and the assumption that the *known* value of a property for one substance can be used to estimate the unknown value of the same property for another substance. This assumption also implies that the potency of an effect shared by different analogous substances is similar and is also usually the result of an expert judgement evaluation.

A chemical category is comprised of a group of chemicals which the physicochemical, toxicological, and ecotoxicological properties are expected to be comparable due to their structural similarity. These structural similarities may provide a predictable pattern in physicochemical properties, environmental fate, environmental effects, and human health effects. The similarities of chemical groups in a category can be based on a common functional group (e.g., aldehyde, ketone, ester, etc.), an incremental change across the category (e.g., a methylene group change in the chain length), or the common precursors or breakdown products due to physical or biological processes resulting in a structurally similar substances (e.g., family approach of related chemicals, such as carboxylic acid, ester etc.). Within a category, various substances may be selected for the endpoint desired. If the available test results demonstrate that the chemical substances in a category are behaving similarly, then interpolation, extrapolation, or both can be used to fill the data gaps of the target chemical.

Read-across techniques are widely used as part of US EPA’s Pre-Manufacture Notification Process and High Production Volume program [17–20], as well as in the New Chemical Categories document [21] used to review new chemicals under the Toxic Substances Control Act. The US EPA’s Office of Pollution Prevention and Toxics, whose experience with read-across approaches is relatively mature, has developed the Analog Identification Methodology and the Chemical Assessment Clustering Engine, specifically designed to assist in reviewing and prioritizing large inventories of chemicals and to facilitate read-across and data gap-filling for untested substances. In addition, about 75% of REACH (Registration, Evaluation, Authorization and Restriction of Chemicals) dossiers contain data derived from read-across for at least one endpoint [22]. Approximately 150,000 of the 850,000 documents used for the REACH registrations were based on read-across/grouping approaches [23].

It has been well documented the read-across process is very widely used in toxicity estimation of various chemicals [24–26] and nanomaterials [27–29]. One of the main reasons for employing read-across, or other new approach methodologies (NAMs) to identify/evaluate any adverse effects relevant to human health, is these NAMs will not require any animals for testing/evaluation. For example, recently Ahlers et al. [30] have applied read-across to assess the hazard and aquatic toxicity of thio-based chemicals to fulfill the REACH requirements. Abe et al. [31] developed a read-across workflow for skin irritation and corrosion predictions using the Organisation for Economic Co-operation and Development Quantitative structure-activity relationship (OECD QSAR) toolbox and the use of metabolomics to support read-across has been published with phenoxy herbicides [32], and aminoalcohols [33]. Patlewicz et al. have recently reviewed [9,34] the various available read-across tools for application in toxicity prediction. In the area of emission and release in the manufacturing phase, read-across has been applied in the estimation of indoor air guidance values [35], hazard assessment [36], and exposure assessment [37,38]. The read-across process has also been employed for the estimation of emission release factors for the manufacture and use of adhesives and sealants using the emission release factors of coatings and paints [39]. Given all the documented applications of read-across techniques, its application to estimation of emissions from a manufacturing chemical unit process is a natural fit. This is similar in approach to toxicity prediction when applied to emissions estimation.

## 3. Chemical process emissions and estimation

In the chemical industry, thousands of chemical substances are manufactured and used in various applications. These chemical production processes consist of a multitude of chemical reactions and a number of unit process operations, such as extraction, isolation, and purification to name a few. In addition, a variety of chemical processing aids such as catalysts, solvents, and reagents are employed in the manufacturing process in different types of equipment. Thus, in chemical industrial processes various types of chemicals are emitted/released into the environment during the manufacturing of the target product and during each chemical’s use. Environmental releases include emitting, spilling, leaking, pumping, pouring, emptying, discharging, injecting, escaping, leaching, dumping, or disposing into the environment for any chemical or chemical mixture. The term “environment” includes water, air, and land, the three media to which a release may occur [40]. Some of the air emission sources are (1) fugitive releases, (2) point sources, such as equipment venting and incomplete separation, (3) residuals from containers, such as drums, tank trucks and rail cars, and (4) equipment cleaning. There are two types of air emissions, (1) Primary emissions, which are direct emissions from the chemical process and include unit process emissions and fugitive emissions. (2) Secondary emissions are greenhouse gases resulting from heating processes, such as combustion of fossil fuels in boilers. The main sources of fugitive emissions are leaks from valves, pressure relieve valves, open ended lines and sampling connections, compressor and pump seals and building ventilation systems. Point sources include unit operations equipment such as reactors, absorption columns, distillation columns, condensers, and dryers. Point source releases normally include air pollution control equipment and the emissions take place through vents, stacks, and pipes. Emissions will also occur during cleaning of vessels and process equipment. Various factors can influence the rate and magnitude of emissions and release from a chemical process. These include the type of reaction, type of process, process conditions, equipment used, and the physicochemical properties of the substances used in the process, etc. As mentioned earlier chemical manufacturing emissions can be estimated through various approaches, and a variety of estimation methods are reported in literature for quantifying the environmental releases [41] from a chemical manufacturing process.

Two alternate approaches in estimating and quantifying the releases of a chemical during its manufacturing life cycle stage were reported by US EPA’s Office of Research and Development. The first approach is a top-down methodology [42] which utilizes EPA databases such as the National Emissions Inventory and Toxics Release Inventory to obtain air emissions, water discharges, and releases to land data based on the information submitted to US EPA by the manufacturing industry. The second approach is a bottom-up process design and simulation method [5], which utilizes modeling to calculate input-output flows of materials and emission factors to estimate air emissions. The US EPA’s Air Pollutant Emission Factors handbook [4] provides a compilation of air pollution estimates, or emission factors for approximately 200 air pollution source categories using source test data, material balance, and engineering calculations. When there is no emission data available one can utilize emission scenario documents (ESDs) developed by OECD member countries or use US EPA’s Chemical Screening Tool for Exposures and Environmental Releases (ChemSTEER) to identify relevant data.

Although direct measurements provide the most reliable emission information, they are expensive and time-consuming methods to implement for every process. Herein we propose the use of read-across applications to estimate the emissions for a specific chemical based on the available data of an analogous chemical or a set of chemicals in a representative category. Thus, if environmental release data for a set of analogous chemicals in a category are available, one can utilize read-across to fill in the data gaps either by interpolation or extrapolation. In the absence of monitored emission data, one can utilize the estimated emission data in a read-across process. However, the confidence levels of this read-across data depends on the reliability of the estimates itself.

## 4. Application of read-across approach for emissions estimation

As described earlier read-across can be performed in an analogue approach or category approach [11] and accomplished in four different manners to fill the data gaps. These four manners are, (1) one-to-one (one analogue used to make an estimation for a single chemical); (2) one-to-many (one analogue used to make estimations for two or more chemicals); (3) many-to-one (two or more analogues used to make an estimation for a single chemical); (4) many-to-many (two or more analogues used to make estimation for two or more chemicals). When the available data are limited, the one-to-one analogue approach is typically seen as the only possible option [11,13].

The analogue approach is used when the process is carried out using a single structurally similar chemical, where there is no trend or regular pattern among the properties. The simplest example of an analogue approach is carrying out a read-across process from one single source substance to a target substance. When the analogue approach utilizes more than one substance, then the process needs to be repeated for each source and target substances.

In a category approach, read-across is done among several structurally similar chemical substances. Due to structural similarity the toxicological, ecotoxicological and/or environmental fate properties and human health effects of a chemical category are expected to be similar or follow a regular pattern. The source and target chemical substances should have the same functional groups, and the alkyl chain lengths of adjacent source chemicals should not differ by more than 2 carbon atoms and differences in physicochemical properties should not influence toxicological properties [12].

Several resources are available to support the use of read-across, and for determining chemical similarity [9,16]. Various tools and databases are available to estimate structural similarity including EPA’s Analog Identification Methodology tool [43], the OECD QSAR Toolbox and ToxRead [9].

**Figure 1.**
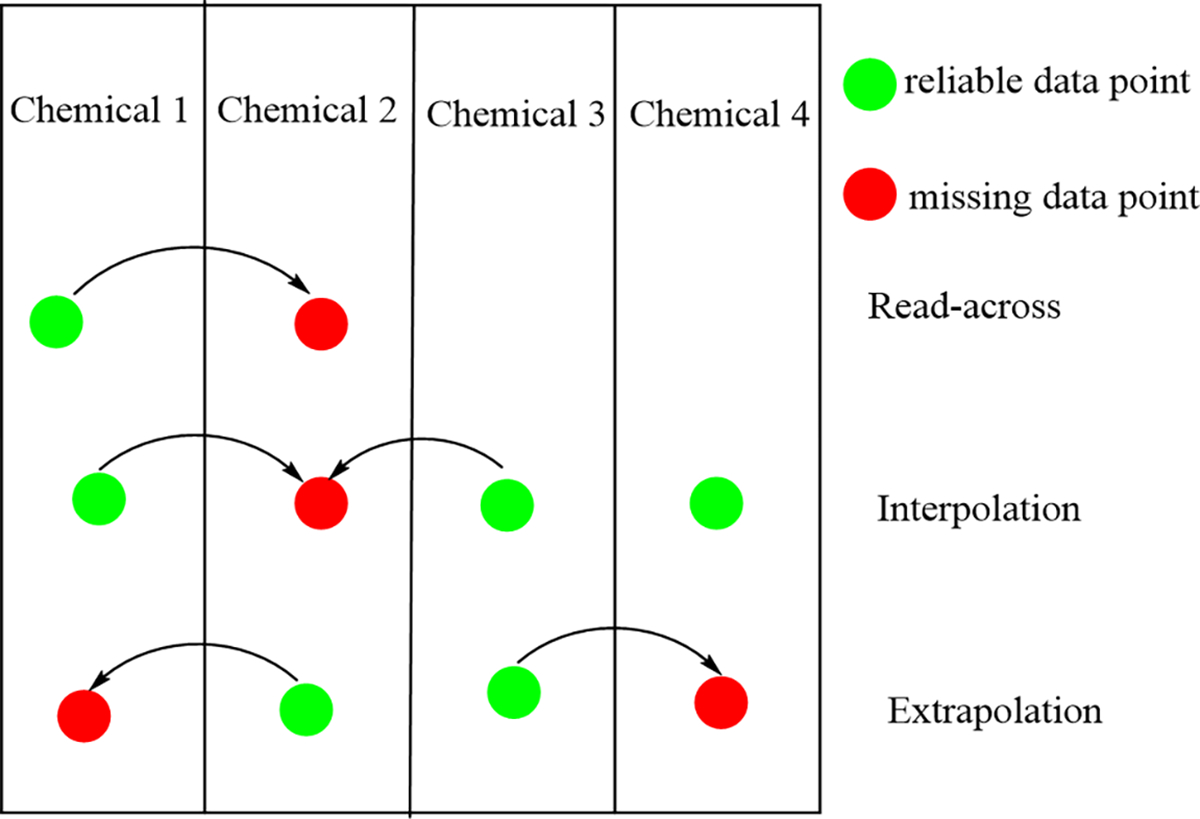
Read-across techniques to filling data gaps.

Within a category, various substances may be selected for the endpoint desired. If the available test results demonstrate that the chemical substances in a category are behaving similarly, then interpolation, extrapolation or both can be used to assess the target chemicals. When no regular pattern is observed for the property under consideration in a category of chemicals, the target chemical property may be estimated based on a read-across from a category member with relevant information (Figure 1). A chemical category assessment approach has an advantage when consistent patterns are observed in the available data within a category, which increases confidence in the reliability of the target values for all the individual chemicals in that category [10].

Recently Luechtefeld et al. [44] have advanced the read-across approach by developing a large database containing 833,844 property values for about 80,908 chemicals and applying read-across structure activity relationship approaches *via* machine learning techniques to estimate the hazard of a chemical utilizing the known hazard data of chemical analogs. This development has prompted the authors to envision a similar scenario with chemical emissions from various chemical manufacturing processes and use.

To develop and apply a read-across approach for manufacturing emission/release prediction, the authors envision the construction of a database with available environmental release data from various databases. Sources for this database can be comprised with appropriate information from the Toxics Release Inventory, National Emissions Inventory, European Chemicals Agency, and others. This database can be linked to EPA’s CompTox Chemicals Dashboard, PubChem, ChemSpider, and others to obtain a chemical’s physicochemical and toxicological properties. However, the available environmental release data and the physicochemical properties of chemicals will also have many data gaps. It is hoped that these data gaps can be filled using additional read-across and property estimation methods combined with expert knowledge. This can be accomplished for a small number of defined data gaps. However, when there are several data gaps in the available data, developing and applying machine learning processes would be more appropriate.

In this contribution, we propose extending the use of read-across methodology into the estimation of chemical emissions for a chemical process (where data are not available) by reading across the available emission/release data of an analogous chemical in a similar manufacturing process. Read-across methodology can also be extended to chemical categories when emission data are available for certain chemicals within a category. The application of this read-across process and its reliability depends on the similarity of the source and target chemical’s physicochemical properties such as molecular weight, vapor pressure, boiling point, melting point, water solubility and partition coefficient, and the source and target chemical processes. This also includes measures taken to reduce/eliminate pollution (control technologies), for example using air filters to capture the chemical dust/mist in the air. The type of equipment used, as well as process temperature and reaction time will play a crucial role. Obviously, the reliability of the read-across estimated data depends on the quality of the source emission data itself. The high-quality measured source data provides a dependable target estimated emission data assuming that there is a very high degree of similarity in all the criteria relevant to the chemical process.

The framework for application of a read-across methodology for chemical process emissions estimation can be viewed as follows:

- Problem identification: First step in the process is identification of the target chemical and the corresponding chemical manufacturing process for which chemical emissions are to be estimated.
- Characterization of target chemical process: The characterization of the target chemical process involves collecting all relevant available data such as structural, physicochemical properties of target chemical, reactants and the auxiliary chemicals used in the chemical process and the production volume. In addition, information regarding chemical process conditions and pollution prevention approaches should also be included.
- Selection of structural analog source chemicals/chemical processes and their characterization: In the selection process of source chemical compounds, first a set of structurally similar compounds need to be identified. Web-based structure searching tools typically include an algorithm to search for structurally similar chemicals with a Tanimoto similarity cut off. Tanimoto coefficient is a measure of the number of common substructures shared by two molecules. A Tanimoto index of 1 indicates both molecules are identical, whereas a Tanimoto index of 0 indicate that two molecules have nothing in common [45]. Similarity searches for chemicals are provided in common online tools such as ChemID plus (https://chem.nlm.nih.gov/chemidplus/), Chemspider (http://www.chemspider.com/StructureSearch.aspx) as well as in commercial applications such as SciFinder. In addition, The Analog Identification Methodology tool [43] developed by SRC Inc for US EPA’s OCSPP can be used to identify potential analogues. In the data collection process for structurally similar source chemicals, the same data types as for the target chemical should be included along with production volumes. For example, physicochemical properties of the source chemical, reactants and auxiliary chemicals, process reaction temperature and time and pollution prevention approaches. Since the read-across process is dependent on experimentally derived data it is critical that some of the selected source compounds have available measured emission data.
- Data gap analysis for target and source chemicals and data gap filling (e.g., any missing physicochemical properties): Physicochemical property information can be obtained from the PubChem database or CompTox Chemicals Dashboard. If the data is not available for a particular chemical, physicochemical properties can be estimated from its chemical structures and quantitative structure-property relationships [46]. A number of tools are available for predicting physicochemical properties such as open structure-activity Relationship App (OPERA), Estimation Programs Interface [7], and Online Chemical Database (OCHEM).
- Compare the chemical processes at target and source situations and assumptions made.
- Carryout the read-across process for target chemical process emissions based on source chemical process emissions.
- Determine if another iteration is necessary to reduce the number of data gaps, increase the accuracy of the initial values, and evaluate assumptions made.

When the difference in the target and source chemical properties and process conditions are very small, one can assume that the emissions will be similar from both the processes. However, if the differences in the physicochemical properties and reaction process conditions (temperature and time) are somewhat different, one can generate a read-across factor for each property and derive an overall read-across factor [38]. For conducting a quantitative read-across process, OECD suggests the use of four possible approaches for predicting the outcome [11]: (1) Reading across from the endpoint value of a closest source chemical, (2) Applying a trend analysis if data are available for two or more source chemicals, (3) Processing the end point value of two or more source chemicals by averaging the value, (4) When enough data are available within a category of chemicals, considering the most conservative value of the source chemicals. Alternatively, when some chemicals have available measured data and follows a certain trend, missing data gaps can be estimated.

- Uncertainty assessment: Dependent on data quality, data relevance, etc. Uncertainty may be described as low, moderate, or high [47]. High data quality and structurally very similar chemicals with very similar processes will have low uncertainty.

**Figure 2.**
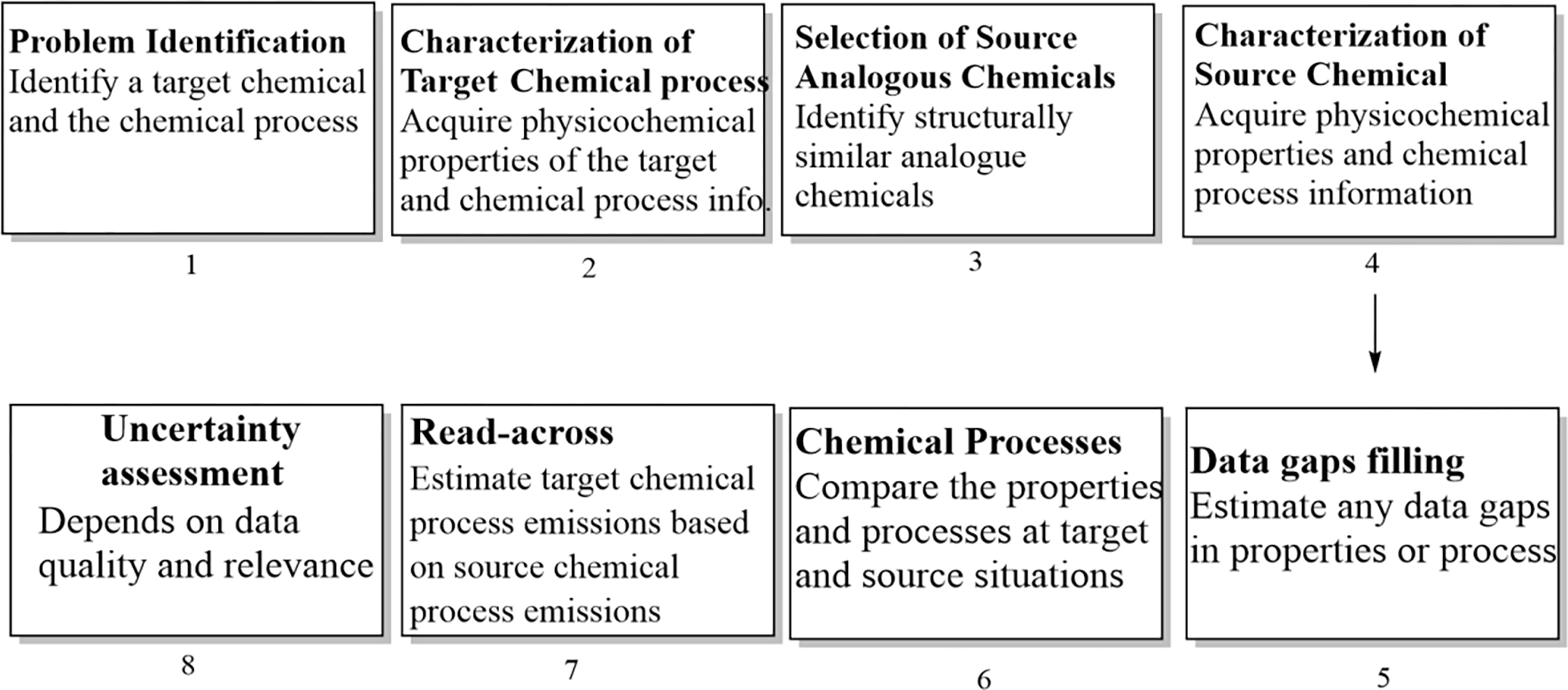
Read-across framework for predicting emissions from a chemical process.

This type of read-across approach can be applied only when the target and source chemical processes are very similar with similar auxiliary chemicals. For example, if the target chemical compound process uses a solid acid catalyst and the source chemical process uses a liquid acid catalyst, it may be difficult to conduct a read-across approach for emission prediction for these two processes. The steps involved in this proposed read-across framework for emission prediction are graphically depicted in Figure 2.

## 5. Read-across example for emission estimation for a chemical process

To apply the read-across framework for emission estimation from a chemical process proposed in this manuscript, an example with a one-to-one read-across approach is undertaken to demonstrate the applicability of the framework. Although a simple one-to-one approach is considered here, this framework can be applied to other methods such as one-to-many, many-to-one, or many-to-many read-across approaches. In the problem identification, *p*-cymene (4-methylcumene) is selected as the target compound. The next step in the framework is to identify a few structurally similar analogues. Structural similarity can be evaluated using diverse structural descriptors and algorithms or by systematic variation of key features [48]. It is critical that some of the selected source compounds have available direct measured emissions data, and this measured data for the source compounds becomes a basis for estimating the emissions of the target compound using read-across [49].

To identify the structurally similar analogues for p-cymene, a search was conducted using the US EPA’s Analog Identification Methodology tool, as well as Royal Society of Chemistry’s ChemSpider. Both tools identified cumene as the closest analogue of our target chemical p-cymene. ChemSpider identified cumene with a Tanimoto similarity index of more than or equal to 99%. In addition, both tools identify isomeric compounds, as well as diisopropyl and triisopropyl benzenes as analogous compounds. ChemSpider also identified 4-ethylcumene as an analogue with a Tanimoto similarity index of more than or equal to 99%. Some of the analogue compounds identified for toxicity purposes are assumed to degrade or metabolize to the target compound so that metabolites and target compounds might have similar toxicity. However, when it comes to emissions, this molecular structural difference might make a huge difference because the physical properties change considerably.

**Figure 3.**
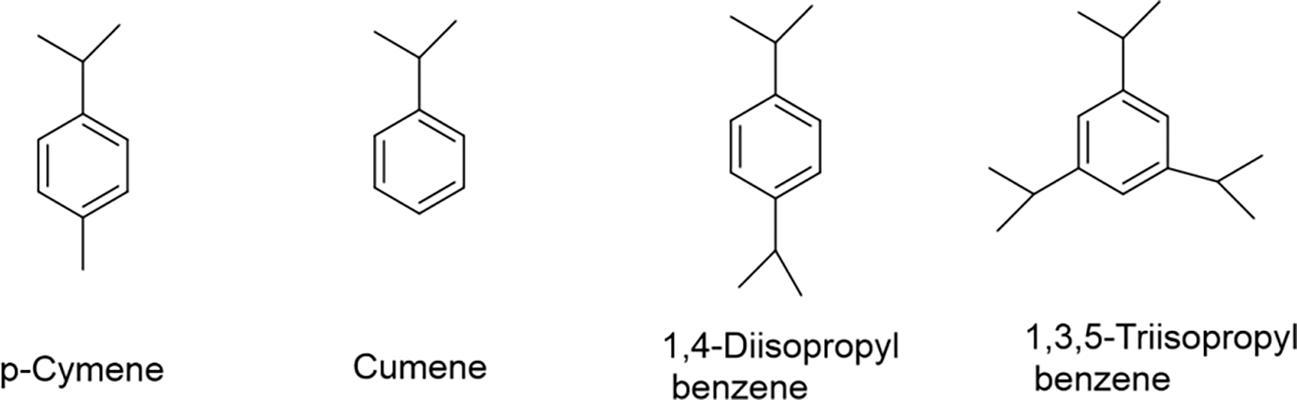
Chemical structures of target and source chemicals.

After identifying the structurally similar analogues (chemical structures of source analogue chemicals are shown in Figure 3), or source chemicals, of the target compound, collection of the physicochemical properties of the target chemical and source chemical is performed. Table 1 provides some of the physicochemical properties of analogous alkylbenzenes which are obtained from PubChem database. This list of properties provided here is intended for presentation purposes. The list can be as extensive as desired to provide sufficient data for the read-across evaluation.

When directly compared the physicochemical properties of the identified analogue cumene to the target compound p-cymene, cumene source chemical’s molecular weight, boiling point and vapor pressure values are closely similar to those for *p*-cymene. Cumene is the most closely related structurally analogue chemical with a pendant isopropyl group attached to benzene but is lacking a methyl group in the para position when compared to the target compound.

**Figure 4.**
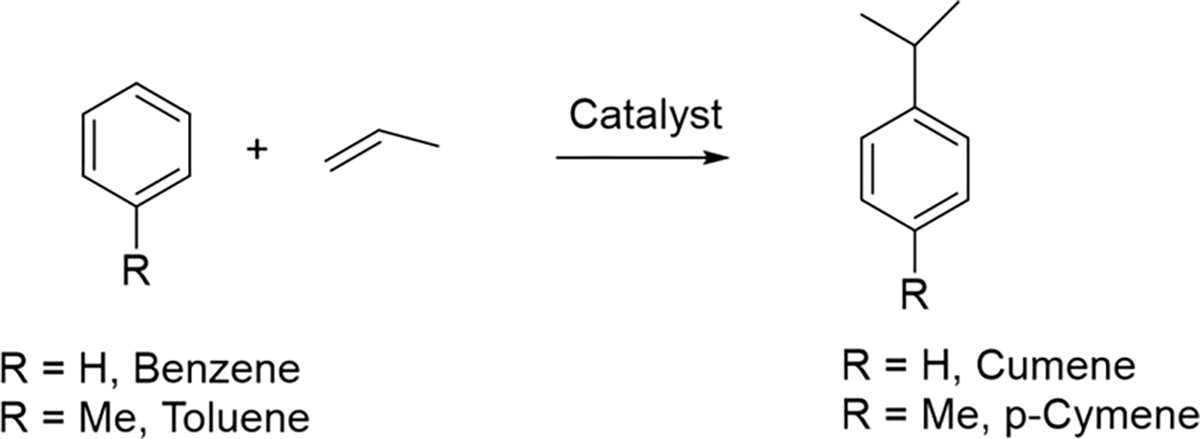
Synthesis of cumene and p-cymene.

Next, the manufacturing chemical process information used for the source compound and the target compound were obtained to compare each manufacturing process. As mentioned in the earlier section, it is imperative to have very similar chemical manufacturing processes to apply the read-across approach for emission estimation. The *p*-cymene [50] and cumene [51] manufacturing processes are very similar to one another, each utilizing a solid acid-catalyzed alkylation reaction step with propylene gas. The chemical reaction scheme is depicted below in Figure 4. Thus, it is appropriate to utilize the emission data of cumene to read-across for estimating the emissions of p-cymene process. Read-across extrapolates the measured emission data of the source compound cumene to the target compound p-cymene.

In the cumene manufacturing process, benzene (reactant and solvent), propylene (reactant), and cumene (product) are expected to be the primary chemical emissions (Table 2). For the purposes of demonstration, we are utilizing the emissions data that is reported in the literature for the manufacturing process of cumene [41,52].

The reactants used in the manufacturing process of *p*-cymene are toluene and propylene. Thus, the emissions expected from this process are toluene, propylene, and *p*-cymene. The main difference in the reactants of these two processes is, the cumene process uses benzene, whereas *p*-cymene process uses toluene (Table 3). Since the differences in the molecular weight and properties of these two chemicals is not significant, one can extrapolate using the read-across from cumene to *p*-cymene and the emissions are expected to be very similar from both the processes.

If one would like to consider the differences in the properties, a read-across factor for each property can be calculated by dividing the value at target situation by its value at source situation and the overall read-across factor can be used to estimate the emissions [38].

For the sake of application of this methodology and approach, this example is one of the most simplistic from a reaction synthesis and manufacturing standpoint. The overall scheme is Reactant A plus Reactant B over a solid acid catalyst to form Product C, with by-products of unreacted starting materials, which can be separated with non-specialized separation process unit operations.

While the results are identical for this simplified example, we expect them to partially hold for the two chemicals (target and source) given the high degree of similarity between them. Additionally, the assumption of the two chemical processes being near identical also lends to this high degree of similarity in results. This can be viewed as one end of the spectrum, and the other being a chemical emission/release comparison being one with stark differences between the target and source chemical properties, their manufacturing, downstream processing, and pollution control unit operations. While the intent is to identify scenarios for target and source chemical and their manufacturing to be as similar as possible, this will not always be the case.

Even with this simplified example, the need for measured data for the source chemical was difficult to obtain and ensure the data used is representative of the process under evaluation. While it would be near impossible to have the entirety of data across the manufacturing, use, and end-of-life phases for a source chemical at this time, this is the goal we should strive for to generate and evaluate true and accurate emissions/releases from a chemical across these life cycle stages. As the structural complexity of the chemical increases, so will the manufacturing process, and the need for data grows exponentially.

As discussed in an earlier section, data comes in many forms and with an appropriate level of certainty or uncertainty. For this application of data, along with many others, the order of preference for data is, measured which is the most representative, followed by data obtained from read-across method, then data gathered from simulations, then estimated data, and finally data from rule of thumb, which does have some trend to it, but is usually the least representative. Throughout the development of this framework, it was evident there is a significant lack of available measured data for manufacturing processes.

EPA research over the last 10 years has demonstrated the ability to evaluate chemical manufacturing processes for material usage, waste generation, utility consumption, emissions/releases, and sustainability evaluation. Each of these are tools and methods that will be applied in the further development of this framework. These efforts span the range from synthesis route identification and ontology [52], process unit simulation and ontology [53], exposure estimation and ontology [54], data mining [55], emission quantification from the bottom-up [41] and top-down methodology [5], end-of-life modeling [55], industrial process system assessment [56], and sustainability evaluation [57].

The combination of these tools will allow the establishing of a framework to assemble a representative chemical manufacturing process for not only the source, but also the target chemical. With the ability to have a greater volume of data which is better in representing the actual process a higher level of estimation of emissions/releases can be obtained. The goal is developing a gate-to-gate life cycle inventory (LCI) of a chemical manufacturing process to support the application of life cycle assessment in the design, evaluation, and regulation of sustainable chemicals.

## 6. Conclusions

This work contributes to the development of read-across applications and methods, which are data gap filling approaches, for advancing the development of tools for the estimation of emissions/releases. The envisioned application of read-across applications in this contribution can aid in the population of data gaps between source and target chemicals in the areas of physical properties, manufacturing processes, downstream process unit operations and end-of-life pathways. The culmination of this vast wealth of data, can not only allow for the evaluation and estimation of the emissions and releases attributed to a chemical under consideration for approval and manufacturing in the US, but it can also lend itself for outward applications in being able to facilitate evaluations across a chemical family or manufacturing approach. By leveraging data usage, this application can incorporate features into models associated with the manufacturer, industry sector, molecular descriptors, support to environmental policy development and decision making, and economic impact (positive and negative) to the commercial sector.

Future work for this application will explore the development of a more extensive case study to detail sources of data from measured to rule of thumb, translation of collected data into direct application for this framework, the use of existing EPA methods and tools for the development of representative data for manufacturing, downstream unit operations, and pollution control technologies, and creating a data infrastructure to house the collected and curated data.

## Figures and Tables

**Table 1.** Physicochemical properties of p-cymene and cumene.

Chemical compound	p-Cymene	Cumene	1,4-Diisopropyl benzene	1,3,5-Triisopropyl benzene

Mol. weight	134.2 amu	120.2 amu	162.27 amu	204.35 amu
Boiling point	176.8 °C	152.4 °C	210.3 °C	232 °C
Vapor pressure	1.55 mm Hg 25 °C	4.6 mm Hg 25 °C	0.24 mm Hg 25 °C	0.02 mm Hg 25 °C
Water solubility	23.4 mg/L	50 mg/L	Insoluble in water	Insoluble in water
Log K_OW_	4.10	3.66	NA	NA

**Table 2.** Emissions from cumene production.

Chemical emission	Air emission Kg/Kg of cumene production

Benzene	5.9E-06
Propylene	7.8E-06
Cumene	1.9E-05

**Table 3.** Emissions from p. cymene production.

Chemical emission	Air emission Kg/Kg of p-cymene production

Toluene	5.9E-06
Propylene	7.8E-06
p. Cymene	1.9E-05
